# Involvement of ATF6 in Octreotide-Induced Endothelial Barrier Enhancement

**DOI:** 10.3390/ph17121604

**Published:** 2024-11-28

**Authors:** Saikat Fakir, Nektarios Barabutis

**Affiliations:** School of Basic Pharmaceutical and Toxicological Sciences, College of Pharmacy, University of Louisiana Monroe, Monroe, LA 71201, USA

**Keywords:** lung injury, ARDS, inflammation, somatostatin

## Abstract

**Background/Objectives**: Endothelial hyperpermeability is the hallmark of severe disease, including sepsis and acute respiratory syndrome (ARDS). The development of medical countermeasures to treat the corresponding illness is of utmost importance. Synthetic somatostatin analogs (SSA) are FDA-approved drugs prescribed in patients with neuroendocrine tumors, and they act via growth hormone (GH) suppression. Preclinical investigations suggest that Octreotide (OCT) alleviates Lipopolysaccharide (LPS)-induced injury. The aim of the study is to investigate the involvement of activating transcription factor 6 (ATF6) in the protective effects of OCT in endothelial dysfunction. To the best of our knowledge, the available information on that topic is limited. **Methods**: Human lung microvascular endothelial cells (HULEC-5a) and bovine pulmonary artery endothelial cells (BPAEC) which expressed elevated levels of ATF6 due to AA147 were exposed to OCT or vehicle. Protein expression, endothelial permeability, and reactive oxygen species (ROS) generation were assessed utilizing Western blot analysis, Fluorescein isothiocyanate (FITC)-Dextran assay, and Dichlorofluorescein diacetate measurements, respectively. **Results**: Our observations suggest that ATF6 activation significantly improves OCT-induced endothelial barrier enhancement. This combination led to increased expression of binding immunoglobulin protein (BiP) and glucose-regulated protein 94 (Grp94), which are downstream unfolded protein response (UPR) targets. Moreover, ATF6 activation prior to OCT treatment resulted in decreased activation of myosin light chain 2 (MLC2) and cofilin; and reduced reactive oxygen species (ROS) generation. ATF6 activation enhanced the anti-inflammatory effects of OCT, as reflected in the suppression of transducer and activator of transcription (STAT) 1, STAT3, and P38 phosphorylation. **Conclusions**: Our findings suggest that ATF6 activation prior to OCT treatment enhances the beneficial effects of OCT in the endothelium.

## 1. Introduction

The endothelial barrier integrity is essential for maintaining vascular homeostasis, controlling the exchange of fluids, proteins, and immune cells between the bloodstream and surrounding tissues [1]. Dysregulation of this barrier function is a hallmark of various pathological conditions, including acute lung injury (ALI) [2], acute respiratory distress syndrome (ARDS) [3], and sepsis [4]. Multiple factors contribute to endothelial barrier disruption, including pro-inflammatory cytokines ((e.g., tumor necrosis factor alpha (TNF-α), interleukin-1 beta (IL-1β)), bacterial endotoxins (e.g., Lipopolysaccharides), and reactive oxygen species (ROS) generation [5,6,7]. These stimuli promote cytoskeletal rearrangement, cell contraction, and compromise intercellular junctions between endothelial cells. The present study demonstrates for the first time the involvement of activating transcription factor 6 (ATF6) in the effects of Octreotide (OCT) in the inflamed endothelium.

Somatostatin modulates neurotransmission in the central nervous system and controls the secretion of growth hormone and thyrotropin [8]. OCT, a synthetic somatostatin analog (SSA), is involved in the inhibition of growth hormone (GH) secretion and cell proliferation. OCT synthesis, as well as its pharmacological activities, was reported in 1982 by Bauer et al. [9]. This 14 amino-acid cyclic peptide exerts its effects by binding to somatostatin receptors (SSTRs) that are expressed in various tissues, including endothelial cells [10]. OCT can ameliorate endothelial and lung injury due to LPS, introducing the possibility that it may be useful for disorders related to endothelial leak and disruption (e.g., sepsis, ARDS) [11,12].

Unfolded protein response (UPR) is activated upon accumulation of misfolded or unfolded proteins within the endoplasmic reticulum (ER) [13]. This cell stress response mechanism is mediated by three primary signaling pathways, each of which is activated by a distinct sensor protein: inositol-requiring enzyme 1 alpha (IRE1α), protein kinase RNA-like ER kinase (PERK), and ATF6 [14]. Upon endoplasmic reticulum (ER) stress, ATF6 is transported to the Golgi apparatus, where it is subjected to proteolytic cleavage. Then, it is translocated to the nucleus to upregulate genes involved in protein folding, ER-associated degradation (ERAD), and lipid biosynthesis [15]. Activation of this transmembrane protein protects against various forms of cell challenges, including oxidative stress, inflammation, and hypoxia [16].

In endothelial cells, ATF6 activation can enhance the expression of factors (i.e., heat shock proteins) involved in maintaining endothelial integrity [17]. These proteins assist in the proper folding of nascent proteins and prevent the aggregation of misfolded cell elements, thereby supporting cell survival and function during stress. Moreover, ATF6 has been shown to modulate inflammatory signaling pathways, including the nuclear factor kappa beta (NF-κB) and mitogen-activated protein kinase (MAPK) pathways, which are closely linked to endothelial barrier dysfunction [18].

In recent years, there has been growing interest in identifying therapeutic agents and molecular pathways related to vascular function and hyperpermeability-related diseases. ATF6 activation represents a promising therapeutic target for improving barrier function in conditions characterized by ER stress and inflammation, such as lung injury. The present study suggests for the first time that targeted ATF6 activation enhances OCT-induced barrier enhancement; utilizing measurements of paracellular permeability, ROS generation, and assessment of major inflammatory marker activation.

## 2. Results

### 2.1. AA147 and OCT Induce BiP in Endothelial Cells

**Figure 1:**
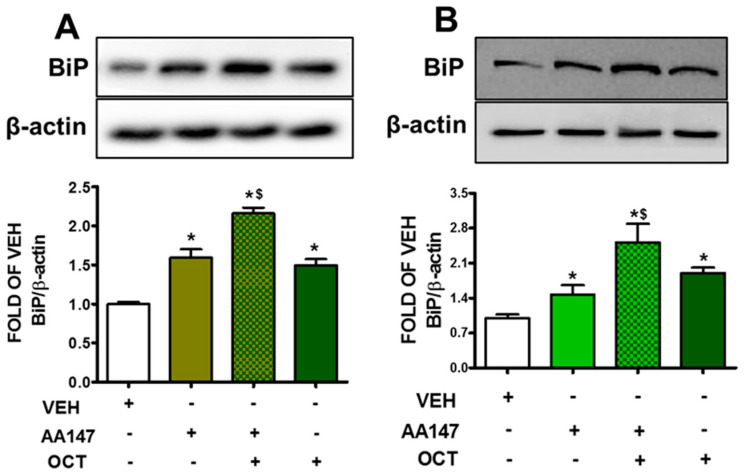
Western blot analysis of BiP induction in BPAEC (**A**) and HULEC-5a (**B**) treated with vehicle (0.1% DMSO) or AA147 (10 μM) for 24 h; prior to exposure to vehicle (0.1% DMSO) or OCT (300 nM) for 24 h. Signal intensity was analyzed by densitometry. Protein levels of BiP were normalized to β-actin. The blots shown are representative of three independent experiments. * *p* < 0.05 vs. VEH, and ^$^ *p* < 0.05 vs. AA147. Means ± SEM.

BPAEC (Figure 1A) and HULEC-5a (Figure 1B) treated with AA147 (10 μM, 24 h) were exposed to OCT (300 nM, 24 h), and the corresponding vehicles. Our observations suggest that AA147 as well as OCT induce BiP, and that effect was greater in the endothelial cells which were exposed to both reagents.

### 2.2. AA147 Enhances OCT-Induced Grp94 Augmentation in Endothelial Cells

**Figure 2:**
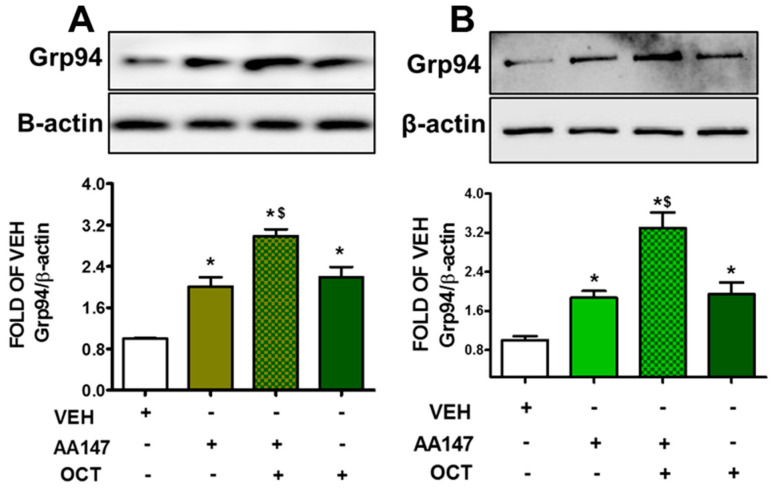
Western blot analysis of Grp94 activation in BPAEC (**A**) and HULEC-5a (**B**) treated with vehicle (0.1% DMSO) or AA147 (10 μM) for 24 h; prior to exposure to vehicle (0.1% DMSO) or OCT (300 nM) for 24 h. Signal intensity was analyzed by densitometry. Protein levels of Grp94 were normalized to β-actin. The blots shown are representative of three independent experiments. * *p* < 0.05 vs. VEH, and ^$^ *p* < 0.05 vs. AA147. Means ± SEM.

BPAEC (Figure 2A) and HULEC-5a (Figure 2B) treated with AA147 (10 μM, 24 h) were exposed to OCT (300 nM, 24 h), and the corresponding vehicles. Our observations suggest that both AA147 and OCT affect Grp94 expression, a downstream target of ATF6. Furthermore, the cells which were exposed to both AA147 and OCT presented with higher levels of Grp94 expression, as compared to the cells exposed to AA147 or OCT, alone.

### 2.3. ATF6 Enhances OCT-Induced Cofilin Deactivation in Endothelial Cells

**Figure 3:**
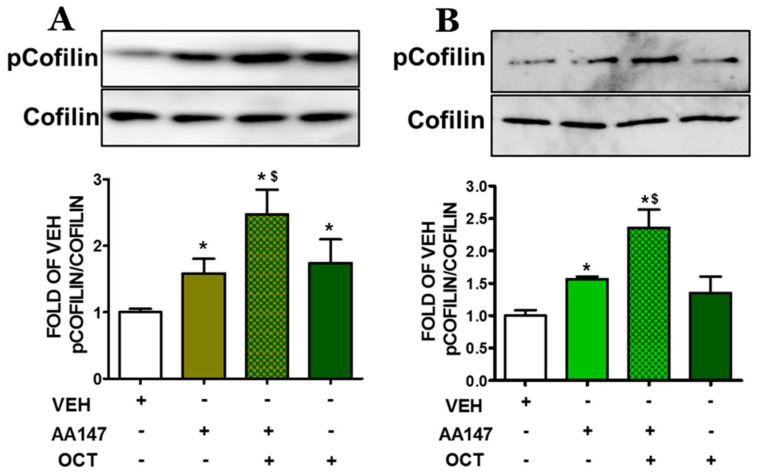
Western blot analysis of phosphorylated Cofilin (pCofilin) and Cofilin in BPAEC (**A**) and HULEC-5a (**B**) exposed to either VEH (0.1% DMSO) or AA147 (10 μM) (24 h); and post-treated with either VEH (0.1% DMSO) or OCT (300 nM) (24 h). The blots shown are representative of three independent experiments. Signal intensity was analyzed by densitometry. * *p* < 0.05 vs. VEH, and ^$^ *p* < 0.05 vs. AA147. Means ± SEM.

AA147 enhances OCT-induced phosphorylation of Cofilin (pCofilin) in both BPAEC and HULEC-5a cells. In BPAEC (Figure 3A), and HULEC-5a cells (Figure 3B), pre-treatment with AA147 (10 μM) for 24 h followed by a 24 h exposure to OCT (300 nM) significantly increased pCofilin levels as compared to vehicle-treated cells.

### 2.4. ATF6 Enhances OCT-Induced MLC2 Deactivation in Endothelial Cells

**Figure 4:**
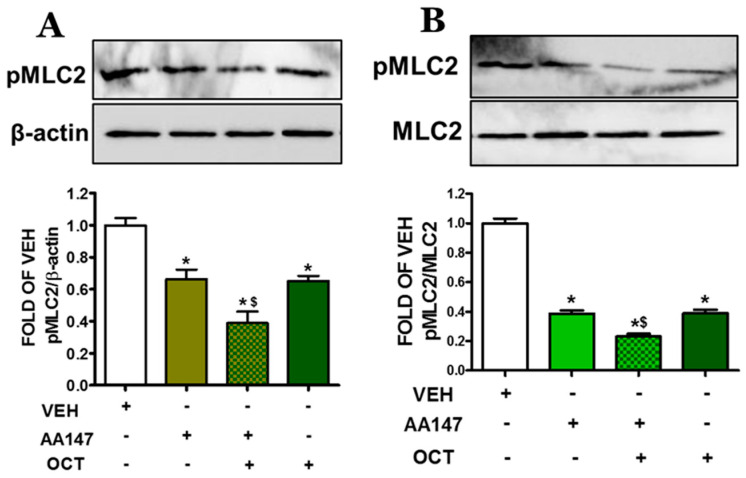
Western blot analysis of phosphorylated MLC2 (pMLC2) in BPAEC (**A**) and HULEC-5a (**B**) treated with VEH (0.1% DMSO) or AA147 (10 µM) for 24 h, and then post-treated with either VEH (0.1% DMSO) or OCT (300 nM) (24 h). Signal intensity was analyzed by densitometry. The blot shown is representative of 3 independent experiments. * *p* < 0.05 vs. VEH, and ^$^ *p* < 0.05 vs. AA147. Means ± SEM.

AA147 enhances OCT-induced MLC2 dephosphorylation in both BPAEC and HULEC-5a cells, as demonstrated by Western blot analysis. In BPAEC (Figure 4A), and HULEC-5a cells (Figure 4B), pretreatment with AA147 (10 μM) for 24 h, followed by a 24 h exposure to OCT (300 nM), significantly reduced pMLC2 levels as compared to vehicle-treated cells.

### 2.5. ATF6 Enhances OCT-Induced Barrier Enhancement in Endothelial Cells

**Figure 5:**
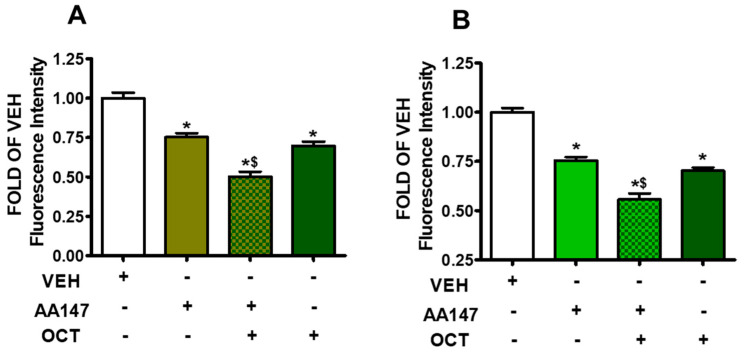
Assessment of paracellular permeability utilizing FITC-Dextran in BPAEC (**A**) and HULEC-5a (**B**) exposed to vehicle (0.1% DMSO) or AA147 (10 μM) for 24 h; and post-treated with vehicle (0.1% DMSO) or OCT (300 nM) for 24 h. * *p* < 0.05, vs. VEH, ^$^ *p* < 0.05 vs. AA147, n = 5. Means ± SEM.

AA147 enhances OCT-induced barrier integrity in both BPAEC and HULEC-5a cells, as assessed by the FITC-Dextran permeability assay. In BPAEC (Figure 5A) and HULEC-5a cells (Figure 5B), pretreatment with AA147 (10 μM) for 24 h, followed by a 24 h OCT (300 nM) exposure, significantly enhanced barrier function, as compared to vehicle-treated cells. Both AA147 and OCT treatment reduced paracellular permeability. The cells pretreated with AA147 prior to OCT demonstrated better barrier function, as compared to the cells which were treated with OCT or AA147 alone.

### 2.6. AA147 Enhances OCT-Induced ROS Suppression in Endothelial Cells

**Figure 6:**
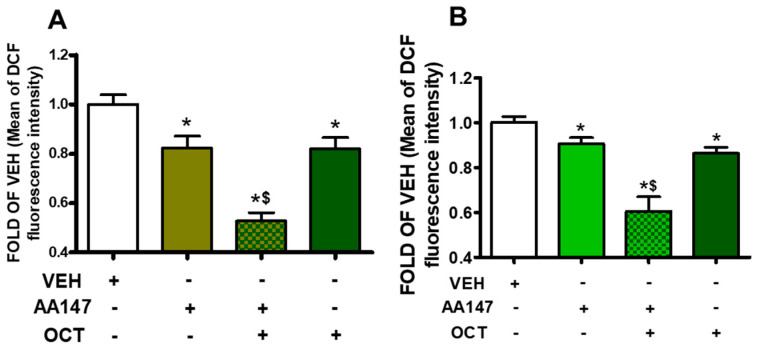
BPAEC (**A**) and HULEC-5a (**B**) were exposed to vehicle (0.1% DMSO) or AA147 (10 μM) for 24 h; and were post-treated with vehicle (0.1% DMSO) or OCT (300 nM) for 24 h. ROS were measured with the DCFDA cellular ROS detection assay kit. * *p* < 0.05 vs. VEH, and ^$^ *p* < 0.05 vs. AA147; n = 4 per group (Panel **A**) and n = 8 per group (Panel **B**). Means ± SEM.

AA147 and OCT reduce reactive oxygen species (ROS) production in both BPAEC and HULEC-5a cells, suggesting that both compounds have anti-oxidative functions. In BPAEC (Figure 6A) and HULEC-5a cells (Figure 6B), ATF6 activation prior to OCT resulted in a greater reduction in ROS generation, as compared to AA147 or OCT treatment alone.

### 2.7. ATF6 Enhances OCT—Induced STAT1 Suppression in Endothelial Cells

**Figure 7:**
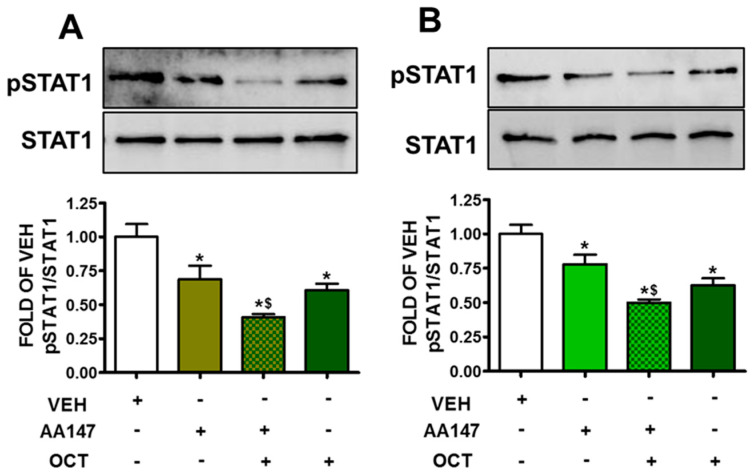
Western blot analysis of phosphorylated STAT1 (pSTAT1) and STAT1 in BPAEC (**A**) and HULEC-5a (**B**) treated with VEH (0.1% DMSO) or AA147 (10 µM) for 24 h, and then post-treated with either VEH (0.1% DMSO) or OCT (300 nM) (24 h). Signal intensity was analyzed by densitometry. The blot shown is representative of 3 independent experiments. * *p* < 0.05 vs. VEH, and ^$^ *p* < 0.05 vs. AA147. Means ± SEM.

BPAEC (Figure 7A) and HULEC-5a (Figure 7B) were pre-treated with AA147 (10 μM) for 24 h, prior to a 24 h exposure to OCT (300 nM). AA147 and OCT treatment resulted in decreased phosphorylated STAT1 (pSTAT1) levels compared to vehicle-treated cells, and presented synergistic effects.

### 2.8. ATF6 Enhances OCT—Induced pSTAT3 and pP38 Suppression in Endothelial Cells

**Figure 8:**
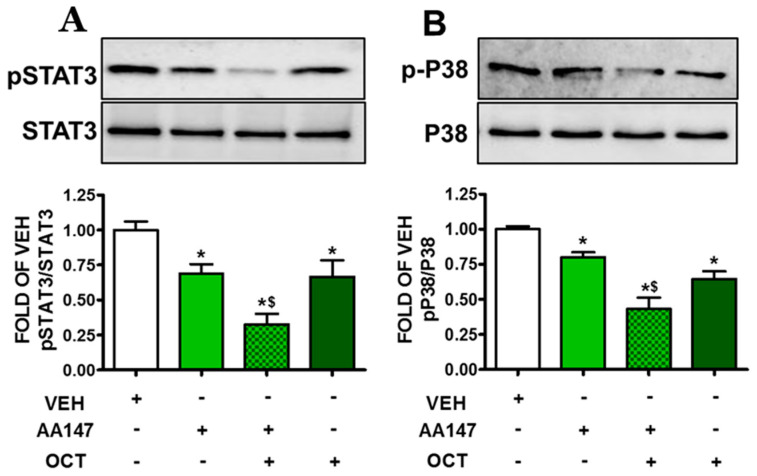
Western blot analysis of phosphorylated STAT3 (pSTAT3) and STAT3 (**A**); and phosphorylated P38 (p-P38) and P38 (**B**) in HULEC-5a treated with VEH (0.1% DMSO) or AA147 (10 µM) for 24 h, and then post-treated with either VEH (0.1% DMSO) or OCT (300 nM) (24 h). Signal intensity was analyzed by densitometry. The blot shown is representative of 3 independent experiments. * *p* < 0.05 vs. VEH, and ^$^ *p* < 0.05 vs. AA147. Means ± SEM.

Human lung microvascular endothelial cells treated for 24 h with AA147 (10 μM) were exposed for 24 h to OCT (300 nM). The results shown in (Figure 8A) suggest that both compounds suppress the activation of STAT3, and activation of ATF6 prior to OCT treatment resulted in greater pSTAT3 suppression. Similar observations in the context of p-P38 are observed in (Figure 8B).

## 3. Discussion

Endothelial barrier dysfunction is associated with several critical diseases including ALI, ARDS, and sepsis [19,20]. SSA such as OCT are in clinical practice for the treatment of acromegaly and neuroendocrine tumors (Table 1). OCT has emerged as a promising candidate for restoring endothelial barrier integrity. This somatostatin analog exerts protective effects on the vasculature by modulating inflammation, reducing oxidative stress, and enhancing endothelial function [11]. However, the precise molecular mechanisms underlying the barrier-enhancing properties of OCT remain poorly understood. One key pathway that may interact with OCT to augment its therapeutic effects is the ER stress response, particularly UPR [21]. ATF6 is a UPR sensor which plays a central role in maintaining cellular homeostasis under conditions of stress by modulating the expression of proteins involved in folding, trafficking, and quality control within the ER [22].

ATF6 has been implicated in the protection against a variety of stressors in endothelial cells. However, its role in regulating endothelial barrier function, particularly in conjunction with OCT, remains largely unexplored [17]. The present study reveals that ATF6 activation enhances the barrier-enhancing effects of OCT, presenting a promising approach for mitigating endothelial dysfunction in lung inflammatory diseases.

The ATF6 inducer AA147 potentiates the expression of chaperone proteins (i.e., BiP and Grp94) involved in UPR [23,24,25]. The upregulation of BiP (Figure 1) and Grp94 (Figure 2) in endothelial cells exposed to OCT suggests that ATF6 is involved in the protective effects of OCT towards barrier protection [26]. This is particularly relevant in inflammatory environments where endothelial cells face increased oxidative stress and inflammation, leading to protein misfolding. AA147 in combination with OCT leads to a more pronounced activation of the aforementioned UPR markers. Those changes provide the necessary protein-folding capacity to withstand the effects of inflammatory and oxidative stressors that typically compromise endothelial barrier function in lung injury [27].

Cytoskeletal proteins (i.e., MLC2, actin) are key regulators of endothelial barrier function [28]. Upon activation by inflammatory mediators, phosphorylation of MLC2 (Figure 4) leads to actomyosin contraction, which results in the formation of stress fibers and increased cell tension [29]. This causes the disassembly of intercellular junctions, thereby increasing paracellular permeability. The actin-binding protein cofilin (Figure 3) is involved in cytoskeletal remodeling by regulating actin depolymerization and reorganization. Cofilin deactivation was significantly enhanced by AA147 in combination with OCT [30]. That effect suggests that ATF6 activation contributes to stabilizing the actin cytoskeleton, thereby preventing endothelial cell contraction and barrier disruption. In the context of lung injury, therapeutic strategies which aim to stabilize the vascular barrier and prevent excessive permeability are highly desirable. OCT has shown promise in this regard by modulating pathways that affect cytoskeletal dynamics, tight junctions, and responses related to inflammation [31,32].

One of the most critical aspects of endothelial dysfunction in lung injury is the activation of mechanisms which exacerbate vascular permeability and tissue damage. AA147-induced ATF6 activation significantly suppresses the phosphorylation of STAT1 (Figure 7), STAT3 (Figure 8A), and p38 (Figure 8B), which are key mediators of inflammatory responses [33,34]. These transcription factors are involved in the expression of inflammatory cytokines and adhesion molecules which facilitate immune cell infiltration into tissues. By inhibiting these signaling pathways, ATF6 activation reduces the inflammatory burden on endothelial cells.

Oxidative stress is a major factor in endothelial barrier dysfunction, particularly in conditions like ARDS and sepsis [35,36]. ROS can directly damage endothelial cells, promoting cytoskeletal rearrangements and weakening intercellular junctions [37]. The study demonstrates that AA147, in conjunction with OCT treatment, leads to a significant reduction in ROS levels (Figure 6). Hence, ATF6 contributes to mitigating oxidative damage, allowing endothelial cells to maintain their structural integrity. The functional outcome of the molecular changes induced by ATF6 activation is a significant improvement in endothelial barrier integrity [38], as demonstrated by the FITC-Dextran permeability assay (Figure 5). Those effects suggest that the combination of ATF6 activation and OCT treatment strengthens the vascular barrier, reducing the passage of fluids and solutes through the endothelial monolayer. Since increased vascular permeability and edema are major contributors to morbidity and mortality, improvement in barrier function presents a promising therapeutic avenue for treating conditions such as ARDS and sepsis [39,40]. Furthermore, our findings suggest that targeting UPR, particularly through ATF6 activation, could be a novel strategy to protect against disorders related to vascular leak. Growth hormone–releasing hormone antagonists suppress GH and counteract inflammation [41,42,43], in line with OCT. However, they are not FDA-approved [44].

## 4. Materials and Methods

### 4.1. Reagents

Octreotide Acetate (PHR1880-10MG) is available from Millipore Sigma (Burlington, MA, USA). Anti-rabbit IgG HRP secondary antibody (95017-556), anti-mouse IgG HRP-linked whole antibody from sheep (95017-554), nitrocellulose membranes (10063-173), DMSO (25-950-CQC), 2′,7′-dichlorofluorescein diacetate (DCFDA) (10180–506), and RIPA solu-tion (AAJ63306-AP) were bought from VWR (Radnor, PA, USA). Protease inhibitor (AB287909) was acquired from Abcam (Cambridge, UK). AA147 (6759) was purchased from Tocris (Bristol, UK). The p-MLC2 (3674S), MLC2 (8505S), P-p38 (9211S), p38 (9212S), pSTAT1 (9167S), STAT1 (9172S), pSTAT3 (9145S), STAT3 (4904S), p-Cofilin (3313S), Cofilin (3318S), BiP (3183S), and Grp94 (2104S) antibodies were purchased from Cell Sig-naling Technology (Danvers, MA, USA). Corning trans-well cell culture inserts (CLS3470), and FITC-Dextran (46945) were bought from Sigma-Aldrich (St. Louis, MO, USA).

### 4.2. Cell Cultures

BPAEC was purchased from Genlantis (San Diego, CA, USA) and was used to assess the goals of the present study. Those cells were sub-cultured in DMEM (VWRL0101–0500). In that media, we added 10% fetal bovine serum which was purchased from VWR (Radnor, PA, USA). Furthermore, human endothelial cells HULEC-5a were maintained in special media developed for such cells (PCS-100-030), and necessary growth factors were added to it (PCS-100-040). The aforementioned reagents purchased were purchased from ATCC (Manassas, VA, USA). Antibiotics and antimycotics in the form of 1 × penicillin/streptomycin were used to avoid cell contamination, and were acquired from VWR, which is located in Rad-nor, PA. The cells were left to grow at 37 °C, and the humified environment was adjusted per suppliers’ recommendations (5% CO_2_–95% air).

### 4.3. Western Blot Analysis

To separate cell proteins based on their molecular weight, Western Blot analysis was utilized. Sodium dodecyl sulfate (SDS-PAGE) Tris-HCl gels were loaded with an equal amount of proteins in a lysis solution. After the separation was concluded, the proteins were moved to nitrocellulose membranes which were exposed to a solution of 5% milk for an hour. Incubation with primary and secondary antibodies followed. The membranes were exposed to primary antibodies (1/1000) overnight, at 4 degrees Celsius. The working concentration of the secondary antibodies was much lower (1/5000). All distinct protein bands were visualized utilizing a special device, namely the ChemiDoc System from Bio-Rad (Hercules, CA, USA) which is available in our laboratory.

### 4.4. Fluorescein Isothiocyanate (FITC)—Dextran Assay

After endothelial cell treatment, FITC dextran (70 kDa, 1 mg/mL) was added to the trans wells for 45 min. 100 mL of culture media were removed from the appropriate wells. Fluorescence was measured with Synergy H1 Hybrid Multi-Mode Reader from Bio-tek (Winooski, VT, USA). The excitation wavelength was 485 nm, and the corresponding value of the emission was 535 nm.

### 4.5. Densitometry and Statistical Analysis

ImageJ (version 1.53e) software from National Institute of Health (Bethesda, MD, USA) was used to measure protein density, and the data appear as Means ± SEM (standard error of the mean). Differences were calculated with Student’s *t*-test to evaluate significance (*p* < 0.05). To assess and calculate statistics GraphPad Prism (version 5.01) was used. The letter n describes the number of repeats.

## 5. Conclusions

Our study reveals for the first time that ATF6 enhances OCT-induced BiP and Grp94 augmentation, Cofilin and MLC2 deactivation, ROS, STAT1, pSTAT3 and pP38 suppression. Future studies in genetically modified mice will aim to substantiate our observations in vivo and assess the effects of SSA, such as Lanreotide, Pasireotide, Vapreotide, and Seglitide) (Table 1) in barrier dysfunction.

## Figures and Tables

**Table 1:** Effects of synthetic somatostatin analogs (SSA) in disease.

SSA	Target Receptors	Applications
Octreotide	SSTR2, SSTR5	Acromegaly, neuroendocrine tumors, refractory diarrhea
Lanreotide	SSTR2, SSTR5	Acromegaly, neuroendocrine tumors
Pasireotide	SSTR1, SSTR2, SSTR3, SSTR5	Cushing’s disease, acromegaly, neuroendocrine tumors
Vapreotide	SSTR2, SSTR5	Palliative treatment of advanced gastrointestinal and pancreatic neuroendocrine tumors
Seglitide	SSTR2	Experimental use in diabetes and endocrine disorders

## Data Availability

Data are available upon reasonable request.
